# Coordinated regulation of the ESCRT-III component CHMP4C by the chromosomal passenger complex and centralspindlin during cytokinesis

**DOI:** 10.1098/rsob.160248

**Published:** 2016-10-26

**Authors:** Luisa Capalbo, Ioanna Mela, Maria Alba Abad, A. Arockia Jeyaprakash, J. Michael Edwardson, Pier Paolo D'Avino

**Affiliations:** 1 Department of Pathology, University of Cambridge, Tennis Court Road, Cambridge CB2 1QP, UK; 2 Department of Pharmacology, University of Cambridge, Tennis Court Road, Cambridge CB2 1PD, UK; 3 Wellcome Trust Centre for Cell Biology, Institute of Cell Biology, University of Edinburgh, Michael Swann Building, Kings Buildings, Mayfield Road, Edinburgh EH9 3JR, UK

**Keywords:** Aurora B, cell division, centralspindlin, cytokinesis, midbody, membrane remodelling

## Abstract

The chromosomal passenger complex (CPC)—composed of Aurora B kinase, Borealin, Survivin and INCENP—surveys the fidelity of genome segregation throughout cell division. The CPC has been proposed to prevent polyploidy by controlling the final separation (known as abscission) of the two daughter cells via regulation of the ESCRT-III CHMP4C component. The molecular details are, however, still unclear. Using atomic force microscopy, we show that CHMP4C binds to and remodels membranes *in vitro*. Borealin prevents the association of CHMP4C with membranes, whereas Aurora B interferes with CHMP4C's membrane remodelling activity. Moreover, we show that CHMP4C phosphorylation is not required for its assembly into spiral filaments at the abscission site and that two distinctly localized pools of phosphorylated CHMP4C exist during cytokinesis. We also characterized the CHMP4C interactome in telophase cells and show that the centralspindlin complex associates preferentially with unphosphorylated CHMP4C in cytokinesis. Our findings indicate that gradual dephosphorylation of CHMP4C triggers a ‘relay’ mechanism between the CPC and centralspindlin that regulates the timely distribution and activation of CHMP4C for the execution of abscission.

## 1. Introduction

Cytokinesis is a rapid and robust process that ensures the faithful segregation of genomic and cytoplasmic contents into the two nascent daughter cells at the end of cell division. Failure in this final step of cell division leads to the formation of polyploid cells and has been associated with several genetic diseases, including cancer [1,2]. Cytokinesis progresses through a series of sequential steps orchestrated by several proteins and protein complexes in a very precise order. After anaphase onset, the mitotic spindle is reorganized into an array of antiparallel and interdigitating microtubules, called the central spindle. The plus ends of central spindle microtubules overlap in a region called the spindle midzone. Signals from the central spindle and astral microtubules position the cleavage plane and trigger the formation of an actomyosin contractile ring that drives the ingression of a cleavage furrow [1]. Furrow ingression compacts central spindle microtubules and ultimately forms an intercellular bridge, which contains at its centre an organelle, the midbody, composed of several proteins and protein complexes responsible for the proper execution and timing of abscission [3,4]. All these proteins display a very stereotypical arrangement at distinct regions of the midbody, which appears to be crucial for the late stages of cytokinesis [3–6]. Various terms have been used to describe the different regions of the midbody; in this paper, we use the terminology of D'Avino & Capalbo [3]. In short, the midbody can be divided into two major regions: the ‘Flemming body’, which corresponds to the central bulge of the midbody containing overlapping microtubule plus ends and various central spindle and contractile ring components, and the ‘midbody arms’, which represent the regions flanking the Flemming body [3]. One protein complex that plays a key role in the assembly and function of the midbody is the chromosomal passenger complex (CPC) [3,6,7]. This complex, composed of a kinase component, Aurora B, the scaffolding subunit inner centromeric protein (INCENP), Borealin and Survivin, also has a key role in surveying the proper segregation of genomes throughout mitosis [8]. In early mitosis, the CPC is responsible for monitoring and correcting the attachments of microtubules to kinetochores, whereas later in cytokinesis it prevents the final abscission of the two daughter cells in the presence of lagging chromatin at the cleavage site, thereby avoiding the formation of genetically abnormal daughter cells [7,8]. The Snf7 component of the endosomal sorting complex required for transport III (ESCRT-III) has been identified as one the CPC's targets in abscission [9,10]. ESCRT proteins are evolutionarily conserved and known for catalysing membrane fission events in several topologically similar events such as abscission, virus budding and the sorting of receptors into vesicles that bud into the lumen of the endosome, creating multivesicular bodies (MVBs) [11]. The ESCRT-III complex is the core machinery that mediates membrane deformation and fission events during these events [12], and Snf7 components (known as CHMP4 proteins in humans) have been shown to form spiral filaments that appear to remodel and constrict the membrane in order to create the abscission site [13]. The CPC has been proposed to regulate abscission timing through direct interaction with the Snf7 components in both *Drosophila* and humans [9,10]. In human cells, Borealin directly interacts with all three CHMP4 paralogues, CHMP4A, CHMP4B and CHMP4C, and Aurora B phosphorylates the terminal tail of CHMP4C [9,10], a region known to regulate CHMP4C's ability to form polymers and associate with membranes [14]. Based on this evidence, two different models have been proposed for the regulation of CHMP4 proteins by the CPC. Carlton *et al*. [10] proposed that Aurora B phosphorylation promotes CHMP4C translocation to the midbody ring, where this ESCRT-III component inhibits abscission. By contrast, we proposed that CPC controls abscission through inhibition of CHMP4 polymerization and membrane association using two concurrent mechanisms: interaction of its Borealin component with all three CHMP4 proteins and phosphorylation of CHMP4C by Aurora B [9]. These two concomitant events could preclude the formation of the ESCRT-III filaments essential for the formation of the constriction that physically separates the two daughter cells. In this model, CHMP4 proteins can assemble into spiral filaments only after CPC removal from the midbody. Overall, the CPC-mediated regulation of ESCRT-III has been suggested to act as a surveillance mechanism that prevents abscission in the presence of DNA at the cleavage site [9,10,15]. However, molecular evidence in support of either model is still lacking, and the mechanistic details of how the CPC controls CHMP4 proteins in abscission are still unclear. Here, we used atomic force microscopy (AFM) to show that purified CHMP4C binds to highly curved membranes and promotes the closure of membrane gaps. Interaction with Borealin prevents the association of CHMP4C with membranes, whereas Aurora B interferes with CHMP4C's ability to close membrane gaps. Furthermore, we show that two different populations of Aurora B-phosphorylated CHMP4C exist, with distinct localization patterns, and that Aurora B phosphorylation is not required for the assembly of CHMP4C spiral filaments at the abscission site. Finally, we show that CHMP4C interacts directly with the kinesin component of the centralspindlin complex and that this interaction is favoured in the absence of Aurora B phosphorylation. Our findings support a model in which the CPC and centralspindlin cooperate to regulate the activity and localization of CHMP4C during cytokinesis.

## 2. Results

### 2.1. The ability of CHMP4C to bind to and remodel membranes is regulated by the chromosomal passenger complex

**Figure 1.**
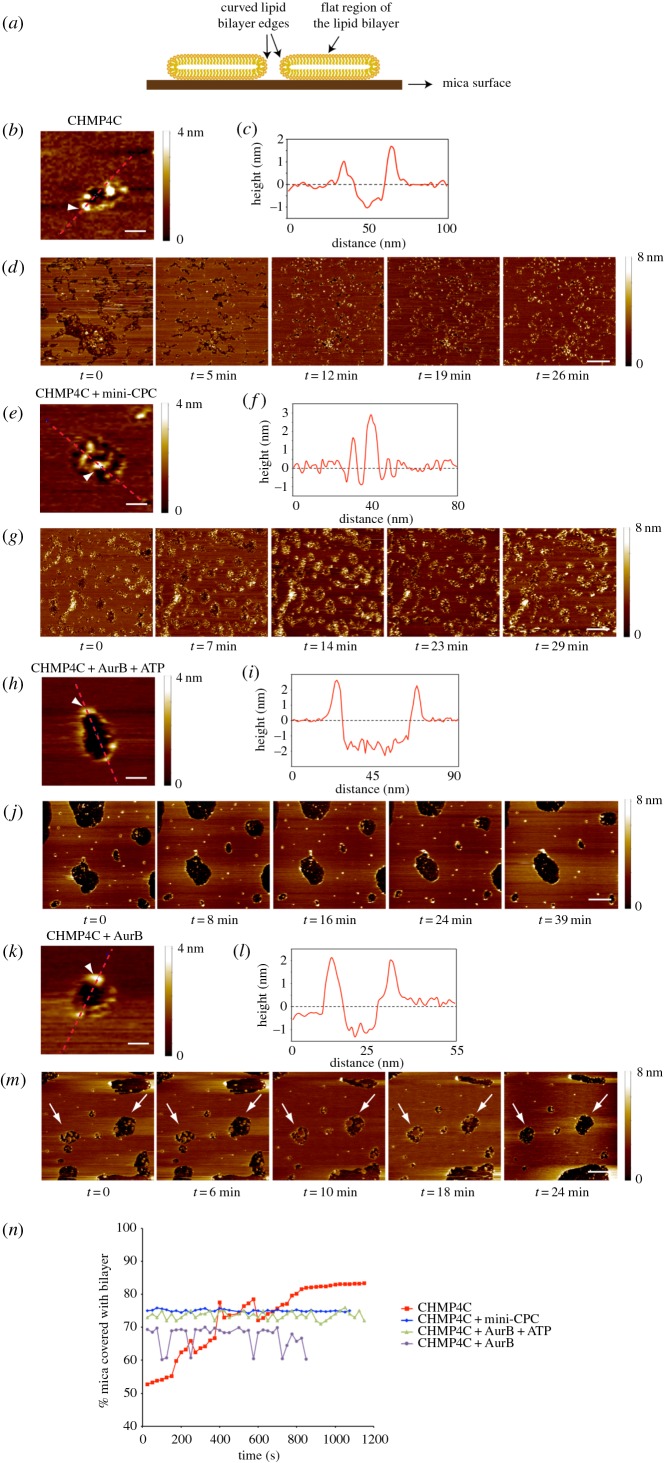
The CPC affects the ability of CHMP4C to bind to and remodel membranes *in vitro*. (*a*) Schematic of a lipid bilayer (yellow) assembled on a mica surface (brown). The central portion of the bilayer is flat, whereas the edges of the bilayer are highly curved. (b) Zoomed AFM image of a lipid bilayer incubated with CHMP4C. A height bar is shown on the right. Scale bar, 20 nm. (*c*) Height relative to the mica surface is plotted along the dotted line shown in (*b*). The position of the arrowhead (at the bilayer edge) along the line is also shown in (*b*), which is raised above the height of the bilayer surface, indicating the presence of protein. (*d*) Sequential AFM images of the same lipid bilayer in the presence of CHMP4C. A height bar is shown on the right. Scale bar, 200 nm. (*e*) Zoomed AFM image of a lipid bilayer incubated with CHMP4C + mini-CPC. A height bar is shown on the right. Scale bar, 50 nm. (*f*) Height relative to the mica surface is plotted along the dotted line shown in (*e*). The position of the arrowhead (at the bilayer edge) along the line is also shown in (*e*), which shows no rise at the bilayer edge, indicating no protein interaction with the bilayer but rather a concentration of protein on the mica surface. (*g*) Sequential AFM images of the same lipid bilayer in the presence of CHMP4C + mini-CPC. A height bar is shown on the right. Scale bar, 200 nm. (*h*) Zoomed AFM image of a lipid bilayer incubated with CHMP4C + Aurora B + ATP. A height bar is shown on the right. Scale bar, 50 nm. (*i*) Height relative to the mica surface is plotted along the dotted line shown in (*h*). The position of the arrowhead (at the bilayer edge) along the line is also shown in (*h*), which is raised above the height of the bilayer surface, indicating the presence of protein. (*j*) Sequential AFM images of the same lipid bilayer in the presence of CHMP4C + Aurora B + ATP. A height bar is shown on the right. Scale bar, 200 nm. (*k*) Zoomed AFM image of a lipid bilayer incubated with CHMP4C and Aurora B. A height bar is shown on the right. Scale bar, 20 nm. (*l*) Height relative to the mica surface is plotted along the dotted line shown in (*k*). The position of the arrowhead (at the bilayer edge) along the line is also shown in (*k*), which is raised above the height of the bilayer surface, indicating the presence of protein. (*m*) Sequential AFM images of the same supported lipid bilayer in the presence of CHMP4C + Aurora B. A height bar is shown on the right. The arrows mark membrane gaps that started closing and then collapsed. Scale bar, 200 nm. (*n*) Variation in the percentage of the mica surface coated with lipid bilayer in the presence of various proteins.

We previously showed that the Borealin central region (residues 110–207) interacts directly with the first 121 amino acids of CHMP4C and that Aurora B phosphorylates this ESCRT-III subunit at three serines in its C terminus: S210, S214 and S215 [9]. To investigate whether the CPC has any role in regulating the ability of CHMP4C to associate with and remodel membranes, we employed AFM, which has already been successfully used to visualize the ESCRT-III machinery on supported lipid bilayers at nanometre resolution [16]. On mica surfaces, without lipid bilayers, CHMP4C alone was visualized as both single particles and filaments (electronic supplementary material, figure S1*a*), indicating that CHMP4C can form polymers *in vitro*. As Borealin cannot be purified alone, we used in our experiments a purified recombinant ‘CPC mini-complex’ (hereafter called mini-CPC), composed only of Borealin, Survivin and the first 58 amino acids of INCENP (thus lacking the phosphorylation module: Aurora B and the INCENP activation segment) [17]. Notably, the presence of Survivin and INCENP_1–58_ will not affect our results because they both interact with the N-terminus of Borealin [17], whereas CHMP4C binds to the Borealin central region [9]. The mini-CPC often appeared as three-pearl strings (electronic supplementary material, figure S1*b*), but when combined with CHMP4C, large globular complexes were observed and no CHMP4C filaments formed (electronic supplementary material, figure S1*c*). These results indicate that binding of CHMP4C to the mini-CPC prevents CHMP4C polymerization, in agreement with our model [9]. On a lipid bilayer composed of 70% l-α-phosphatidylcholine (PC), 15% 1,2-dioleoyl-*sn*-glycero-phosphoethanolamine (DOPE) and 15% 1,2-dioleoyl-*sn*-glycero-3-[phospho-l-serine] (DOPS), CHMP4C filaments assembled specifically at highly curved membranes at the edges of bilayer gaps (figure 1*a–c*). In addition, these filaments appeared to promote the closure of membrane gaps over time (figure 1*d*,*n*; see also electronic supplementary material, movie S1). This behaviour is similar to that observed previously for the *C. elegans* CHMP4B homologue [16], but in our system, human CHMP4C did not require the presence of other ESCRT proteins. We noted that the thickness of a plain bilayer was measured at 4.28 ± 0.13 nm (*n* = 5) (electronic supplementary material, figure S2*a*), similar to what has been previously reported [16], whereas the average apparent thickness of a bilayer after incubation with CHMP4C was approximately 2 nm (electronic supplementary material, figure S2*b*). The decrease in the measured thickness of the bilayer can be attributed mainly to the presence of protein or small lipid fragments in the membrane gaps. Additionally, as the scanning usually continued over several minutes and the membrane gaps began to close, there was a progressive reduction in the measured bilayer thickness, indicating possible thinning/stretching of the bilayer. As expected, the mini-CPC alone did not show any specific membrane association or remodelling activity (data not shown). However, its presence prevented the binding of CHMP4C to the edges of the bilayer gaps and instead large globular complexes accumulated within the gaps (figure 1*e*,*f*). As a result, no membrane remodelling occurred (figure 1*g*,*n*; see also electronic supplementary material, movie S2). These results indicate that the interaction of Borealin with CHMP4C impedes the association of this ESCRT-III component with the membrane, exactly as predicted by our model [9]. We then incubated CHMP4C with active recombinant Aurora B and ATP to promote CHMP4C phosphorylation, and then observed its behaviour on lipid bilayers. In these conditions, CHMP4C was still able to associate with the highly curved membrane at the edges of lipid bilayer gaps (figure 1*h*,*i*), but no progressive gap closure was observed (figure 1*j*,*n*; see also electronic supplementary material, movie S3). Interestingly, after incubating CHMP4C with Aurora B in the absence of ATP, we observed that some membrane gaps began to close, but then collapsed (figure 1*m*,*n*, arrows; see also electronic supplementary material, movie S4). Together, these results indicate that Aurora B phosphorylation inhibits the membrane remodelling activity of CHMP4C, but not its association with highly curved membranes. However, without its kinase activity Aurora B could still partially interfere with CHMP4C activity, which is consistent with the observation that recombinant Aurora B could pull down CHMP4C from cell extracts [10], and with our *in vitro* pull-down assays indicating that bacterially purified GST-tagged Aurora B can interact with *in vitro* translated CHMP4C (electronic supplementary material, figure S3).

### 2.2. Aurora B phosphorylation is not necessary for the formation of CHMP4C spirals at the abscission site

**Figure 2.**
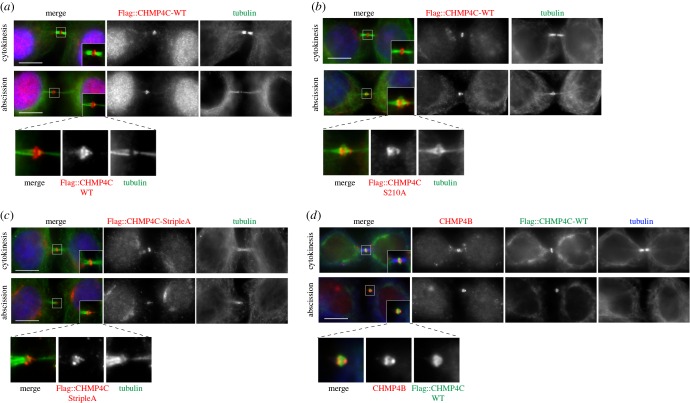
Non-phosphorylatable forms of CHMP4C can form spiral structures at the abscission site. (*a*) HeLa Kyoto cells stably expressing Flag::CHMP4C WT were fixed and stained to detect Flag (red), tubulin (green) and DNA (blue). Insets show two time magnification of the midbody. A three times magnification of the midbody of the cell in abscission is shown at the bottom. Scale bars, 10 µm. (*b*) HeLa Kyoto cells stably expressing Flag::CHMP4C S201A were fixed and stained to detect Flag (red), tubulin (green) and DNA (blue). Insets show two times magnification of the midbody. A three times magnification of the midbody of the cell in abscission is shown at the bottom. Scale bars, 10 µm. (*c*) HeLa Kyoto cells stably expressing Flag::CHMP4C StripleA were fixed and stained to detect Flag (red), tubulin (green) and DNA (blue). Insets show two times magnification of the midbody. A three times magnification of the midbody of the cell in abscission is shown at the bottom. Scale bars, 10 µm. (*d*) HeLa Kyoto cells stably expressing Flag::CHMP4C WT were fixed and stained to detect Flag (green), CHMP4B (red) and tubulin (blue). Insets show two times magnification of the midbody. A three times magnification of the midbody of the cell in abscission is shown at the bottom. Scale bars, 10 µm. In all experiments, the shape and thickness of microtubule bundles were used as criteria to stage telophase cells as described [1,3].

To investigate the role of Aurora B phosphorylation on the ability of CHMP4C to form polymers *in vivo*, we generated HeLa cell lines stably expressing three different Flag-tagged RNAi-resistant versions of CHMP4C: wild-type (WT), a non-phosphorylatable mutant S210A and a mutant that cannot be phosphorylated at any of these three serine residues, S210A, S214A and S215A, dubbed ‘StripleA’. As our previous results indicated that overexpression of all these CHMP4C proteins caused an increase in cytokinesis failure [9], we generated several independent monoclonal cell lines for each construct and specifically selected those that did not show a major increase in the number of multinucleate cells (electronic supplementary material, figure S4). All these Flag::CHMP4C proteins localized to the Flemming body and were able to form spiral filaments at the abscission site (figure 2*a–c*), indicating that preventing phosphorylation of these residues by Aurora B did not affect CHMP4C polymerization and its association with membranes, in complete agreement with our findings *in vitro* (see §2.1). Consistent with this, abscission was not impaired in these cell lines (data not shown). Interestingly, we found that while Flag::CHMP4C proteins always formed a continuous spiral structure that extended from the midbody core to the constriction zone (figure 2*a–d*), CHMP4B showed two distinct localizations, at the Flemming body and at the constriction zone (figure 2*d*), suggesting that different CHMP4 proteins might play distinct roles during abscission.

### 2.3. Two populations of Aurora B-phosphorylated CHMP4C exist with distinct localization patterns during cytokinesis

**Figure 3.**
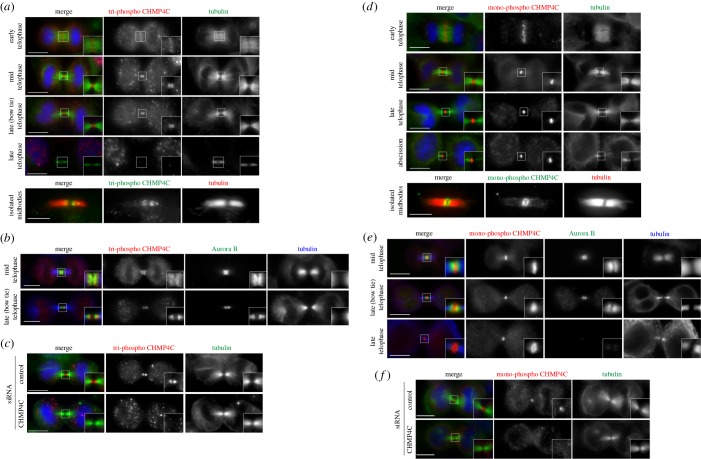
The two phosphorylated forms of CHMP4C display distinct localization patterns during cytokinesis. (*a*) HeLa Kyoto cells were fixed and stained to detect tri-phospho CHMP4C (red), tubulin (green) and DNA (blue). Insets show two times magnification of the midbody. Scale bars, 10 µm. At the bottom, midbodies were purified form HeLa Kyoto cells and fixed and stained to detect tri-phospho CHMP4C (green) and tubulin (red). Scale bar, 5 µm. (*b*) HeLa Kyoto cells were fixed and stained to detect tri-phospho CHMP4C (red), Aurora B (green) and tubulin (blue). Insets show two times magnification of the midbody. Scale bars, 10 µm. (*c*) HeLa Kyoto cells were treated with siRNAs directed against either a random sequence (control) or CHMP4C twice at a 48 h interval and then after 96 h fixed and stained to detect tri-phospho CHMP4C (red), tubulin (green) and DNA (blue). Insets show a two times magnification of the midbody. Scale bars, 10 µm. (*d*) HeLa Kyoto cells were fixed and stained to detect mono-phospho CHMP4C (red), tubulin (green) and DNA (blue). Insets show two times magnification of the midbody. Scale bars, 10 µm. At the bottom, midbodies were purified form HeLa Kyoto cells and fixed and stained to detect mono-phospho-CHMP4C (green) and tubulin (red). Scale bar, 5 µm. (*e*) HeLa Kyoto cells were fixed and stained to detect mono-phospho CHMP4C (red), Aurora B (green) and tubulin (blue). Insets show two times magnification of the midbody. Scale bars, 10 µm. (*f*) HeLa Kyoto cells were treated with siRNAs directed against either a random sequence (control) or *CHMP4C* twice at a 48 h interval and after 96 h fixed and stained to detect mono-phospho CHMP4C (red), tubulin (green) and DNA (blue). Insets show a two times magnification of the midbody. Scale bars, 10 µm. In all experiments, the shape and thickness of microtubule bundles were used as criteria to stage telophase cells as described [1,3].

Our previous data indicated that phosphomimetic CHMP4C mutants did not behave differently from their phosphodead counterparts with respect to their localization and effect on cytokinesis [9], suggesting that they do not reflect the localization and function of phosphorylated CHMP4C. Therefore, to elucidate the role of CHMP4C phosphorylation by Aurora B in cells, we generated two CHMP4C phospho-specific antibodies: one that specifically recognized the form phosphorylated at all three residues, S210, S214 and S215 (tri-phospho) [9], and another specific for the form phosphorylated only at S210 (mono-phospho). These antibodies were extensively validated to show that they recognize only their specific phosphorylated variant (electronic supplementary material, figure S5) [9] and that their signals disappear after depletion of CHMP4C (3). These phospho antibodies displayed different localization patterns during cytokinesis. We found that in metaphase, the tri-phospho CHMP4C antibody accumulated at the plus ends of kinetochore microtubules (electronic supplementary material, figure S6). A signal was also observed on centrosomes, but was not specific because it did not disappear after CHMP4C depletion (electronic supplementary material, figure S6 and data not shown). After anaphase onset, tri-phospho CHMP4C localized to the spindle midzone in early telophase, accumulated at the midbody arms after furrow ingression, and then disappeared in late telophase after the ‘bow tie’ stage (figure 3*a*) [1,3]. This spatial and temporal localization pattern during cytokinesis matches exactly that of the CPC, and indeed, tri-phospho CHMP4C and Aurora B perfectly co-localized during telophase (figure 3*b*). By contrast, mono-phospho CHMP4C did not show any specific staining before anaphase onset (data not shown), but localized to the spindle midzone in early telophase and then specifically accumulated at the Flemming body, where it persisted throughout cytokinesis until abscission, even after the CPC disappeared (figure 3*d*,*e*). Immunostaining on isolated midbodies confirmed that tri-phospho CHMP4C accumulated at the midbody arms, whereas mono-phospho CHMP4C formed a ring around the Flemming body (figure 3*a*,*d*). The signals of the two antibodies were specific for CHMP4C because they disappeared after its depletion (figure 3*c*,*f*).

**Figure 4.**
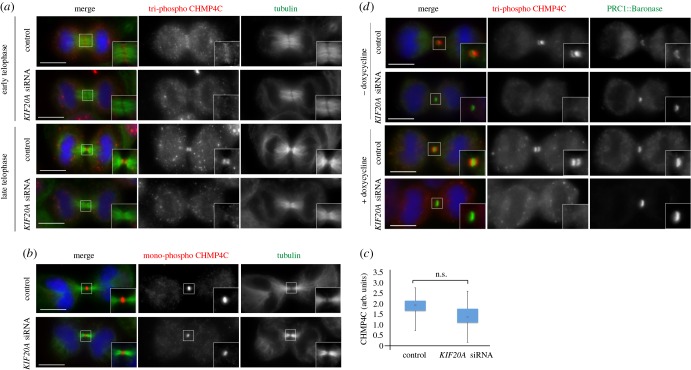
The CPC is required for the localization of tri-phospho CHMP4C at the cleavage site. (*a*) HeLa Kyoto cells were treated with siRNAs directed against either a random sequence (control) or *KIF20A/MKLP2* and after 48 h fixed and stained to detect tri-phospho CHMP4C (red), tubulin (green) and DNA (blue). Insets show two times magnifications of the central spindle and midbody. Scale bars, 10 µm. (*b*) HeLa Kyoto cells were treated with siRNAs directed against either a random sequence (control) or *KIF20A/MKLP2* and after 48 h fixed and stained to detect mono-phospho CHMP4C (red), tubulin (green) and DNA (blue). Insets show two times magnifications of the central spindle and midbody. Scale bars, 10 µm. (*c*) Box plot shows the quantification of mono-phospho CHMP4C fluorescence levels at the midbody calculated as described previously [6]; see also Material and methods. More than 50 cells from three separate experiments were counted for each sample. AU, arbitrary unit. *p* = 0.8 (Student's *t*-test). (*d*) HeLa Kyoto cells carrying a doxycycline-inducible GFP-tagged PRC1-Baronase transgene were treated with siRNAs directed against either a random sequence (control) or *KIF20A* and after 24 h incubated in 2 mM thymidine for a further 20 h. Cells were washed and incubated with or without 1 µg ml^−1^ doxycycline for 10 h and then fixed and stained to detect tri-phospho CHMP4C (red), PRC1-Baronase (green) and DNA (blue). Scale bars, 10 µm. In all experiments, the shape and thickness of microtubule bundles were used as criteria to stage telophase cells as described [1,3].

The perfect co-localization of tri-phospho CHMP4C with the CPC in cytokinesis and the direct interaction between CHMP4C and two CPC components, Borealin and Aurora B (electronic supplementary material, figure S2) [9,10], suggested that the localization of this phosphorylated variant of CHMP4C could depend on the CPC. To investigate the role of the CPC in CHMP4C localization in cytokinesis, we prevented the translocation of this complex to the spindle midzone by depleting the kinesin KIF20A/MKLP2 [18]. No accumulation of tri-phospho CHMP4C at the midbody arms was observed after *KIF20A* RNAi (figure 4*a*), while only a slight reduction of the mono-phospho CHMP4C signal was observed, which was not statistically significant (figure 4*b*,*c*). To determine whether tri-phospho CHMP4C localization required the whole CPC or just Aurora B, we tested if a chimaera composed of the microtubule binding domain of the spindle midzone protein PRC1 coupled with an Aurora B kinase module (dubbed Baronase and comprising a truncated form of Aurora B and the INCENP activating region) [19] could rescue tri-phospho CHMP4C localization after KIF20A depletion. No accumulation of tri-phospho CHMP4C at the midbody arms was observed after KIF20A depletion in cells expressing the PRC1::Baronase chimaera (figure 4*d*), indicating that the localization of this phosphorylated CHMP4C variant to the midbody arms requires the whole CPC and probably its interaction with Borealin.

Together, these results indicate that at least two distinct populations of phosphorylated CHMP4C exist: one pool is phosphorylated at all three serine residues (S210, S214 and S215) at the midbody arms where Aurora B activity is high, whereas the other is phosphorylated only at S210—the strongest phosphorylation site—at the midbody ring, more distant from Aurora B activity. In addition, they also suggest that the localization of tri-phospho CHMP4C at the midbody arms requires the whole CPC and that dephosphorylation at S214 and S215, rather than phosphorylation at S210, may trigger CHMP4C translocation to the midbody ring. It is also noteworthy that neither of the two CHMP4C phospho-antibodies identified the spiral-like structures observed with Flag-tagged CHMP4C proteins, suggesting that phosphorylated CHMP4C is not present in these spiral filaments, which have been proposed to mediate abscission.

Our findings therefore reveal that Aurora B phosphorylation regulates CHMP4C localization, but not its ability to polymerize and form spiral filaments at the abscission site in cytokinesis.

### 2.4. The centralspindlin complex associates preferentially with the low- and unphosphorylated forms of CHMP4C during cytokinesis

**Figure 5.**
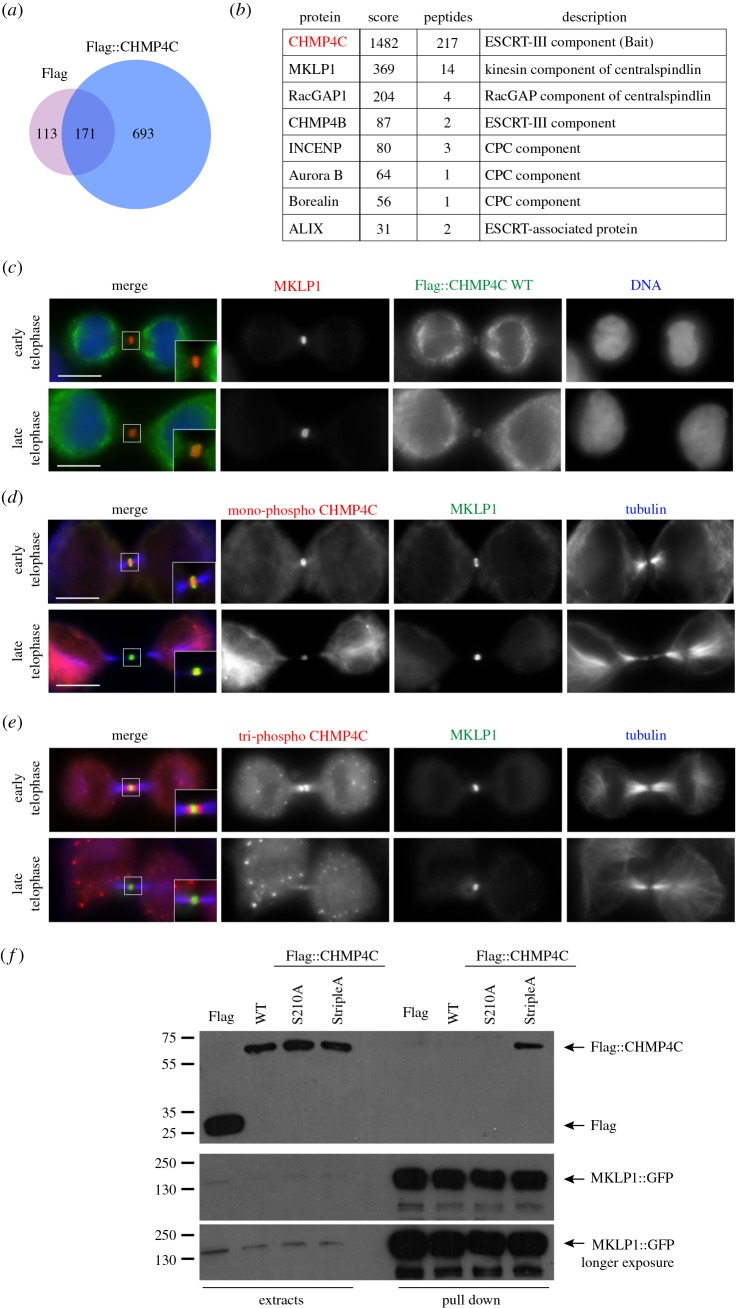
MKLP1 associates with CHMP4C *in vivo*. (*a*) Proportional Venn diagram shows the overlap between the proteins pulled down by the Flag tag alone (284) and the proteins that co-purified with Flag::CHMP4C (864). Note that 693 proteins specifically associated with Flag::CHMP4C. (*b*) Partial list of the CHMP4C-specific interactors identified by affinity purification. A full list can be found in electronic supplementary material, table S1. The bait, CHMP4C, is highlighted in red. (*c*) HeLa Kyoto cells stably expressing Flag::CHMP4C WT were fixed and stained to detect MKLP1 (red), Flag (green) and DNA (blue). Insets show two times magnification of the midbody. Scale bars, 10 µm. (*d*) HeLa Kyoto cells were fixed and stained to detect mono-phospho CHMP4C (red), MKLP1 (green) and tubulin (blue). Insets show two times magnification of the midbody. Scale bars, 10 µm. (*e*) HeLa Kyoto cells were fixed and stained to detect tri-phospho CHMP4C (red), MKLP1 (green) and tubulin (blue). Insets show two times magnification of the midbody. Scale bars, 10 µm. (*f*) HeLa cells stably expressing MKLP1::GFP were transfected with plasmids carrying Flag alone, Flag::CHMP4C WT, Flag::CHMP4C S210A or Flag::CHMP4C StripleA. After 48 h, proteins were extracted and subjected to a pull-down assay using GFP-Trap magnetic beads (ChromoTek). The extracts and pull-downs were analysed by western blot to detect Flag and GFP. The numbers on the left indicate the sizes (kDa) of the molecular mass markers.

The localization of Flag-CHMP4C and mono-phospho CHMP4C to the Flemming body and the requirement of the CPC only for the localization of tri-phospho CHMP4C suggested that other factors might be involved in the dynamic distribution of this ESCRT-III component during cytokinesis. To examine this, we employed our HeLa cell line expressing Flag::CHMP4C (WT) to isolate proteins that associate with CHMP4C during cytokinesis by affinity purification coupled with mass spectrometry (AP-MS). We isolated a total of 693 proteins that were specifically pulled down by Flag::CHMP4C, but not by Flag alone, in HeLa cells synchronized in telophase (figure 5*a*; electronic supplementary material, table S1). Among the isolated proteins, we found, as expected, some previously identified interactors, such as CHMP4B, ALIX (ALG2-interacting protein X, also known as programmed cell death 6 interacting protein, PDCD6IP), and three of the four CPC components—Aurora B, Borealin and INCENP—but also some novel partners, including the two components of the centralspindlin complex, KIF23/MKLP1 and MgcRacGAP/RacGAP1 (figure 5*b*; electronic supplementary material, table S1). Centralspindlin is known to play many essential roles during cytokinesis [20,21], including recruiting the protein Cep55 to the midbody [22], which, in turn, has been reported to be necessary for the recruitment of CHMP4C via the intermediate ALIX [23]. Unexpectedly, Cep55 was not identified in our AP-MS purification, although it is possible that this was simply because of MS limitations. Consistent with the AP-MS findings, we found that MKLP1 co-localized with Flag::CHMP4C and mono-phospho CHMP4C, but not tri-phospho CHMP4C, at the Flemming body in late cytokinesis (figure 5*c–e*). Furthermore, to confirm the interaction between CHMP4C and centralspindlin *in vivo* we performed a reciprocal pull-down experiment by transfecting a monoclonal HeLa cell line stably expressing MKLP1::GFP [24] with Flag::CHMP4C-WT, Flag::CHMP4C-S210A and Flag::CHMP4C-StripleA. Immunoprecipitations using GFP nanobodies indicated that, in synchronized telophase cells, MKLP1::GFP pulled down Flag::CHMP4C-StripleA more efficiently than the other two Flag::CHMP4C proteins (figure 5*f*). Together, these results suggest that, in telophase cells, centralspindlin associates preferentially with the low- and unphosphorylated forms of CHMP4C.

### 2.5. MKLP1 binds directly to CHMP4C and is required for its localization to the midbody

**Figure 6.**
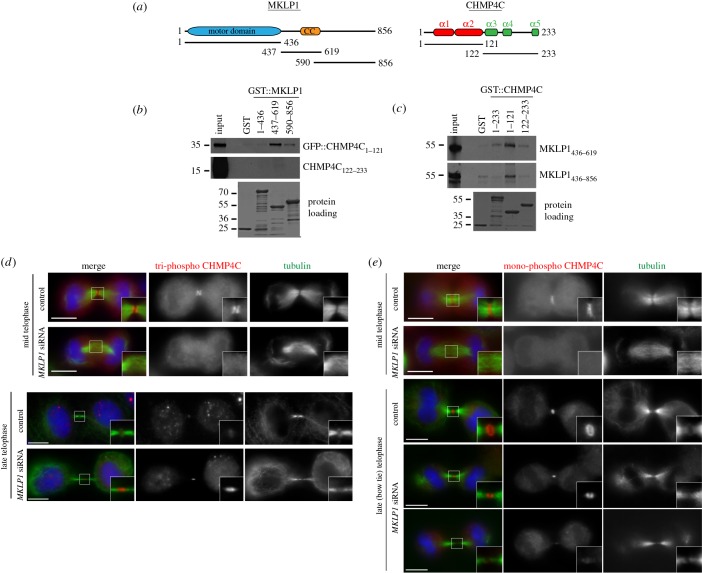
MKLP1 binds directly to CHMP4C and is required for its localization to the central spindle during cytokinesis. (*a*) Schematic diagrams illustrate the protein domains of MKLP1 and CHMP4C. The CHMP4C α-helices are marked at the top. The positions of the different MKLP1 and CHMP4C fragments used for the *in vitro* pull-down assays are also indicated. CC, coiled coil. (*b*) The GST::MKLP1 protein fragments shown at the top and GST alone were incubated with the *in vitro* translated and radiolabelled CHMP4C polypeptides indicated at the right, and then pulled down using glutathione beads. The Ponceau S staining of the protein loading is shown at the bottom and the numbers on the left indicate the sizes (kDa) of the molecular mass markers. (*c*) The GST::CHMP4C protein fragments shown at the top and GST alone were incubated with the *in vitro* translated and radiolabelled MKLP1 polypeptides indicated at the right, and then pulled down using glutathione beads. The Ponceau S staining of the protein loading is shown at the bottom and the numbers on the left indicate the sizes (kDa) of the molecular mass markers. (*d*) HeLa Kyoto cells were treated with siRNAs directed against either a random sequence (control) or *KIF23/MKLP1* and after 48 h fixed and stained to detect triphospho CHMP4C (red), tubulin (green) and DNA (blue). DNA condensation and nuclear shape were used as criteria to stage telophase cells. Insets show two times magnifications of the central spindle and midbody. Scale bars, 10 µm. (*e*) HeLa Kyoto cells were treated with siRNAs directed against either a random sequence (control) or *KIF23/MKLP1* and after 48 h fixed and stained to detect mono-phospho CHMP4C (red), tubulin (green) and DNA (blue). DNA condensation and nuclear shape were used as criteria to stage telophase cells. Insets show two times magnifications of the central spindle and midbody. Scale bars, 10 µm.

To assess whether MKLP1 could directly bind to CHMP4C, we purified recombinant MKLP1 fragments tagged with glutathione-*S*-transferase (GST) from bacteria and tested their ability to pull down *in vitro* translated and radiolabelled CHMP4C polypeptides (figure 6*a*). The N-terminal basic half of CHMP4C, which contains the first two α-helices (aa 1–121), was pulled down very efficiently by the MKLP1 central region containing the coiled coil domain (aa 437–619), and less efficiently by an MKLP1 polypeptide encompassing part of the coiled coil domain and the whole tail region (aa 590–856; figure 6*b*). No interaction was observed between any MKLP1 fragment and the C-terminal half of CHMP4C (figure 6*b*). Reciprocal pull-down assays using GST-tagged CHMP4C fragments confirmed a strong interaction between the N-terminal half of CHMP4C and the C-terminal region of MKLP1 (figure 6*c*).

We then investigated whether MKLP1 was required for the localization of CHMP4C during cytokinesis. Tri-phospho and mono-phospho CHMP4C did not accumulate at the central spindle in *MKLP1* siRNA mid-telophase cells (figure 6*d*,*e*), suggesting that centralspindlin is necessary for the localization of CHMP4C. However, as already known, microtubules failed to assemble into a robust and organized central spindle after MKLP1 depletion (figure 6*d*,*e*), raising the possibility that the absence of CHMP4C at the central spindle could be a secondary, indirect effect. In the few *MKLP1* RNAi cells that managed to complete furrow ingression, tri-phospho CHMP4C persisted much longer than in control cells, although it failed to form two distinct bands (figure 6*d*). By contrast, in these late telophase *MKLP1* RNAi cells, mono-phospho CHMP4C was either absent or failed to form a ring or disc-like structure, its localization appearing similar to that of tri-phospho CHMP4C (figure 6*e*).

To conclude, our results indicate that MKLP1 interacts directly with the N-terminus of CHMP4C and that this kinesin seems necessary for the proper localization of CHMP4C, and in particular for the timely disappearance of tri-phospho CHMP4C and accumulation and correct distribution of mono-phospho CHMP4C in late cytokinesis (figure 6*d*,*e*).

## 3. Discussion

The identification of the CPC as a key factor in surveying abscission in eukaryotes [15,25] and the discovery of the ESCRT-III CHMP4 components as its possible targets in this process [9,10] have begun to unravel some of the mechanisms that regulate the fidelity and timing of the final separation of the two daughter cells during cytokinesis. However, the molecular details are still missing, and our study provides novel crucial insights into this process.

First, our results strongly support and expand our previous model, which posited that the CPC could control the ability of CHMP4C to polymerize and interact with membranes using two concurrent mechanisms [9]. Consistent with our hypothesis, AFM results clearly demonstrated that the presence of a minimal CPC complex, without Aurora B, precluded the interaction of CHMP4C with membranes, whereas Aurora B phosphorylation interfered solely with CHMP4C's membrane remodelling activity (figure 1). Interestingly, Aurora B could also partially inhibit CHMP4C in the absence of its kinase activity, which adds a further level of control of CHMP4C by the CPC.

Second, it is noteworthy that our AFM results also prove, for the first time, that a single CHMP4 protein has the ability to bind to and remodel membranes *in vitro* in the absence of any other ESCRT-III component (figure 1). Our result, together with the observations that CHMP4C accumulates at the cleavage site earlier than other CHMP4 paralogues (figure 3) [10] and is able to assemble into a continuous spiral structure in abscission (figure 2), highlights the uniqueness of CHMP4C. It may also explain why this CHMP4 paralogue is the only one tightly controlled by the CPC via Aurora B phosphorylation [9,10].

**Figure 7.**
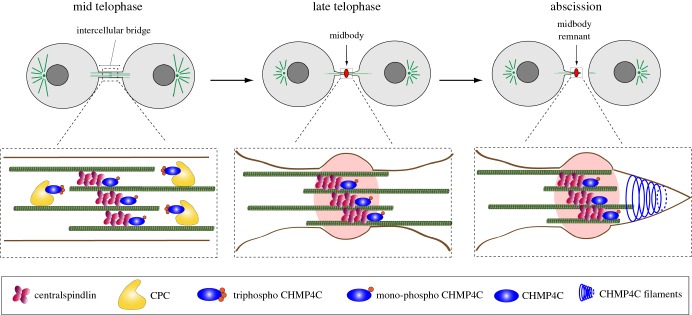
A relay mechanism for the control of CHMP4C by the CPC and centralspindlin. The diagram illustrates the distribution of different forms of CHMP4C and their association with the CPC and centralspindlin at sequential stages of cytokinesis, from mid-telophase to abscission. Full details are given in the text.

Finally, our study identifies two pools of phosphorylated CHMP4C with distinct spatial and temporal localization patterns that appear to depend on their interaction with either the CPC or centralspindlin. Together, our data indicate that centralspindlin *in vivo* associates preferentially with the low- and unphosphorylated forms of CHMP4C, whereas the localization of tri-phospho CHMP4C to the midbody arms depends primarily on the CPC (figures 3–5). Based on these results, we would like to propose that a ‘relay’ mechanism between the CPC and centralspindlin could control the translocation of CHMP4C from the midbody arms to the Flemming body at the end of cytokinesis (figure 7). In this model, at the metaphase–anaphase transition—when Aurora B activity is high and the activity of counteracting phosphatases is low—CHMP4C is bound to the CPC (through its interaction with Borealin) and phosphorylated at all three serine residues by Aurora B. During telophase, the combined increase in phosphatase activity and gradual degradation of the CPC would lead to the progressive de-phosphorylation of CHMP4C, removing first the phosphates at S214 and S215 and then at S210. These changes would release CHMP4C from its association with the CPC and promote its interaction with centralspindlin, causing the translocation of this ESCRT-III component from the midbody arms to the Flemming body, in preparation for abscission (figure 7).

In sum, our findings indicate that CHMP4C plays an active role in membrane deformation during abscission and that its activity and localization are finely regulated by both the CPC and centralspindlin. They do not support the hypothesis that CHMP4C could act as a ‘brake’ by preventing the assembly of a productive ESCRT-III abscission complex [10]. However, the data reported by Carlton *et al*. [10], that CHMP4C depletion induces premature abscission and that Aurora B phosphorylation regulates CHMP4C during abscission, are compatible with our model. Some questions, however, remain unanswered. For example, why did mono-phospho CHMP4C not disappear after preventing the translocation of the CPC to the central spindle and persisted after degradation of the CPC (figures 3 and 4)? One possibility is that phosphorylation at S210 could somehow be protected from the action of phosphatases. Alternatively, CHMP4C could be phosphorylated at S210 also by other kinases that have been described to target ESCRT-III subunits in cytokinesis [26–28]. The identification of the phosphatase(s) that dephosphorylate CHMP4C in late cytokinesis will be important to further confirm and clarify our model. Recent studies have indicated that members of the PP2A family antagonize Aurora B phosphorylation in both metaphase and telophase [29–31]. The catalytic (PP2CA) and regulatory subunit A (PPP2R1A) of PP2A were identified in our AP-MS experiment (electronic supplementary material, table S1), thus making this phosphatase a very strong candidate. However, the catalytic (PPP1CA) and regulatory (PPP1R12A) subunits of the PP1 family were also found (electronic supplementary material, table S1). As PP1 phosphatases have also been shown to antagonize Aurora kinases [32], future investigations are required to fully understand the role of different phosphatases in CHMP4C regulation.

The evidence that MKLP1 interacts with the N-terminal half of CHMP4C, which is known to be required for CHMP4C polymerization and association with membranes [14], suggests that MKLP1 is likely to have the same inhibitory activity as Borealin. This raises the important question of what could be the signal that triggers the release of CHMP4C from MKLP1 and its assembly into the spiral filaments that promote abscission. As a lag exists between the disappearance of the CPC and the formation of CHMP4C spirals in normal conditions (figures 3 and 4) [3,7], we speculate that other signals probably exist in addition to the gradual degradation and inactivation of the CPC. A simple hypothesis could be that CHMP4C release requires the presence of one or more factors that are recruited to the midbody later, just before abscission. Two such factors could be CHMP4B—which has been described to localize to the midbody immediately before abscission [10]—and Cep55, whose interaction with MKLP1 is inhibited by Plk1 phosphorylation until late in cytokinesis [33]. Other proteins known to interact with CHMP4C during cytokinesis, such as ALIX, ANCHR and Vps4 [23,34,35], could also be involved, and future studies will be necessary to integrate all these factors into a comprehensive mechanistic model for ESCRT-mediated abscission.

## 4. Material and methods

### 4.1. Molecular biology and protein expression

Plasmids harbouring CHMP4C non-phosphorylatable mutants have been described previously [9]. The open reading frame (ORF) encoding CHMP4C was amplified by PCR using Accuprime polymerase (Invitrogen) and primers containing *att*B flanking sequences for Gateway technology. The PCR product was purified and introduced into a pDONR221 plasmid (Invitrogen) to generate CHMP4C entry vectors. Then, to generate siRNAi-resistant CHMP4C WT and mutant variants, QuikChange lightning site-directed mutagenesis kit was used (Agilent Technologies). Expression vectors for siRNA resistant Flag::CHMP4C-WT and mutant variants were generated, using recombination with pCMV-Tet02-NtermMycFlag destination vector.

For purification of recombinant CHMP4C, its full-length ORF was cloned into a pET-His6-msfGFP cloning vector (Addgene) as a TEV-cleavable His-tagged protein. The pET-His6-msfGFP-CHMP4C vector was transformed into *Escherichia coli* BL21 Gold competent cells and grown at 37°C in Super Broth-medium containing ampicillin until OD_600_ reached 1. The culture was induced overnight at 18°C with IPTG at a final concentration of 0.35 mM. Cells were resuspended in a lysis buffer (20 mM Tris–HCl pH 8, 750 mM NaCl, 35 mM imidazole and 2 mM 2-mercaptoethanol) and lysed by sonication. The His6-GFP::CHMP4C protein was purified by affinity chromatography using a Ni-NTA column (GE Healthcare). The protein-bound column was washed with lysis buffer followed by 20 mM Tris–HCl pH 8, 1000 mM NaCl, 50 mM KCl, 10 mM MgCl_2_, 2 mM ATP, 35 mM imidazole and 2 mM 2-mercaptoethanol. The protein was eluted with 20 mM Tris–HCl pH 8, 750 mM NaCl, 400 mM imidazole and 2 mM 2-mercaptoethanol. The pooled eluted fractions were dialysed overnight against 20 mM Tris–HCl pH 8, 750 mM NaCl and 2 mM DTT while cleaving with 500 µg of TEV. Concentrated protein was loaded onto a Superdex 200 increase column (GE Healthcare) equilibrated with 20 mM Tris–HCl pH 8, 200 mM NaCl and 2 mM DTT. The purity of the sample was analysed by 15% SDS–PAGE stained with Coomassie blue.

A similar procedure was used to purify the CPC mini-complex (Survivin full-length, Borealin full-length and INCENP 1–57). Borealin was cloned as a TEV-cleavable His-tagged protein in a pETM vector, Survivin as a 3C-cleavable GFP-tagged protein in a pRSET vector (Thermo Fisher) and INCENP 1–57 as an untagged protein in a pMCNcs vector. Proteins were co-expressed in BL21 pLysS with an overnight induction at 18°C. Cells were lysed in 25 mM HEPES pH 7.5, 500 mM NaCl, 25 mM imidazole and 2 mM 2-mercaptoethanol and complex purified by affinity chromatography followed by size exclusion chromatography as above.

### 4.2. Atomic force microscopy

AFM imaging was performed using a Bruker dimension FastScan instrument. All imaging was conducted under fluid, using FastScan D cantilevers (Bruker). Their resonant frequencies under fluid were 110–140 kHz, and the actual scanning frequencies were approximately 5% below the maximal resonance peak. Lipid mixtures containing 70% PC, 15% DOPE and 15% DOPS were dried under nitrogen and hydrated in Biotechnology performance-certified water overnight. Suspensions were probe-sonicated at an amplitude of 10 µA until the mixture became transparent. Liposomes were incubated in the presence or absence of proteins for 30 min and then placed on freshly cleaved mica. In each case, 40 µl of the liposome mixture and an equal volume of buffer (20 mM Tris, pH 8, 100 mM NaCl and 1 mM MgCl_2_) were applied to the mica surface. The mica was washed three times with the same buffer and placed in the fluid cell of the atomic force microscope. The assembled lipid bilayer was immersed in 150 µl of buffer, and all imaging was performed at room temperature. AFM images were acquired at a rate of four frames per minute and plane-fitted to remove tilt. Each scan line was fitted to a first-order equation.

CHMP4C was phosphorylated *in vitro* using recombinant Aurora B (Life Technologies). The assay contained kinase buffer (20 mM HEPES pH 7.5, 5 mM MgCl_2_, 1 mM DTT, 0.1 mM cold ATP) and with or without 190 ng of recombinant AuroraB (Life Technologies). Reactions were incubated at 30°C for 30 min, before incubation with lipids for a further 30 min.

### 4.3. Cell culture, siRNA transfection, drug treatments and generation of stable cell lines

Cell lines stably expressing Flag alone and Flag::CHMP4C constructs were generated by plating 2 × 10^6^ HeLa Kyoto cells in a 100 mm culture dish and transfecting with 19 µg of the appropriate DNA, using FuGENE HD transfection reagent (Promega) for 48 h. Cells were subsequently washed with phosphate-buffered saline (PBS) and cultured in complete selective medium containing 0.4 µg ml^−1^ puromycin for approximately two weeks until colonies became visible. Individual colonies were picked, cultured under resistance and tested for expression of the construct by western blot and immunofluorescence.

HeLa Kyoto cells expressing the PRC1::Baronase chimaera were a gift of F. Barr (University of Oxford) and were maintained in DMEM (Invitrogen) containing 10% fetal bovine serum (FBS, Sigma) and 1% penicillin/streptomycin (Invitrogen) at 37°C and 5% CO_2_. To express the PRC1::Baronase construct, cells were treated with 1 µg ml^−1^ doxycycline for 10 h. The MKLP1::GFP cell line was a gift of M. Mishima (University of Warwick) [24].

For RNA interference, the following siRNAs were used:

scrambled sequence control: 5′-AACGTACGCGGAATACTTCGA-3′;
CHMP4C: 5′-CTCACTCAGATTGATGGCACA-3′;
KIF20A/MKLP2: 5′-AACCACCTATGTAATCTCATG-3′;
KIF23/MKLP1: 5′-GCAGUCUUCCAGGUCAUCU-3′.

Cells were transfected using lipofectamine RNAiMAX (Invitrogen) following the manufacturer's instructions.

### 4.5. Antibodies

CHMP4C phospho-specific antibodies were raised in rabbits against synthetic CHMP4C peptides encompassing residues 206–220 (TARRSRAASSQRAEEC) and containing either a phosphorylated serine at position S210 (TARRpSRAASSQRAEEC; mono-phospho CHMP4C) or phosphorylated serines at position S210, S214 and S215 (TARRpSRAApSpSQRAEEC; triphospho CHMP4C). Sera were first eluted through a column containing a non-phosphorylated peptide, and then each antibody was affinity purified using the appropriate phosphopeptide. Peptide synthesis, conjugation, rabbit immunizations, serum production and affinity purification were carried out by Generon, UK.

Other antibodies used in this study were: mouse monoclonal anti α-tubulin (clone DM1A, Sigma, T9026), chicken polyclonal anti-α-tubulin (Abcam, ab89984), mouse monoclonal anti-Flag (clone M2, Sigma, F3165), rabbit polyclonal anti-Aurora B (Abcam, ab2254), mouse monoclonal anti-Borealin (clone 1D11-MLB Life Science M147-3), rabbit polyclonal anti-KIF23/MKLP1 (clone N19, Santa Cruz Biotechnology, sc-867), rabbit polyclonal anti-CHMP4B, a gift of Sagona & Stenmark [36]. Peroxidase- and Alexa-fluor-conjugated secondary antibodies were purchased from Jackson Laboratories and Thermo, respectively.

### 4.6. Fluorescence microscopy

HeLa cells were grown on glass coverslips (Menzel-Gläser), fixed in ice-cold methanol for 10 min, washed three times for 10 min with PBS and incubated in blocking buffer (PBS, 0.5% [v/v] Triton X-100 and 1% [w/v] BSA) for 1 h at RT. Coverslips were then incubated overnight at 4°C with the primary antibodies, diluted in PBT (PBS, 0.1% [v/v] Triton X-100 and 1% [w/v] BSA). The following day, coverslips were washed twice for 5 min in PBT, incubated with secondary antibodies diluted in PBT for 2 h at RT and then washed twice with PBT and once with PBS. Coverslips were mounted on SuperFrost Microscope Slides (VWR) using Vectashield Mounting Medium containing DAPI (Vector Laboratories). Phenotypes were scored blind and by at least two people independently.

Midbodies were purified form HeLa Kyoto cells as described previously [6,9].

The intensity of mono-CHMP4C fluorescence at midbodies was calculated using the formula: mono-CHMP4C fluorescence = (*I*_CF_ − *I*_C_)/*I*_C,_ where *I*_CF_ is the fluorescence intensity at the midbodies and *I*_C_ represents the background intensity measured within an identical area inside the cytoplasm; more than 50 midbodies from at least three separate experiments were analysed. *p*-values were calculated using Student's *t*-test.

### 4.7. Affinity purification and mass spectrometry

Cells (4 × 10^7^) expressing Flag::CHMP4C WT were synchronized in telophase by a thymidine-nocodazole block-and-release procedure. They were first arrested in S phase by adding 2 mM thymidine (Sigma-Aldrich) for 19 h, washed twice with PBS and released for 5 h in fresh complete medium. After release, cells were cultured for an additional 13 h in fresh complete medium containing 50 ng ml^−1^ nocodazole (Sigma-Aldrich) and then harvested by mitotic shake-off. Mitotic cells were washed three times with PBS, released in fresh medium for 1.5 h and incubated for a further 15 min with the CDK1 inhibitor RO3306 (Calbiochem) at a final concentration of 10 µM. Cells were then harvested by centrifugation and frozen in liquid nitrogen. The cell pellet was resuspended in 5 ml of extraction buffer (50 mM HEPES pH 7.5, 100 mM KAc, 150 mM NaCl, 2 mM MgCl_2_, 1 mM EGTA, 0.5% [v/v] NP-40, 1 mM DTT, 5% [v/v] glycerol and Roche Complete Protease Inhibitors) and homogenized using a high-performance disperser (Fisher). The homogenate was clarified by centrifugation at 750*g* for 20 min at 4°C, and the supernatant was incubated with 200 µl of anti-Flag M2 magnetic beads (Sigma-Aldrich) for 2–4 h on a rotating wheel at 4°C. Beads were then washed four times in 10 ml of extraction buffer for 5 min on a rotating wheel, transferred to a new tube and washed one more time in 10 ml of PBS. Proteins were eluted from beads with 0.5 M NH_4_OH and 0.5 mM EDTA, concentrated, acetone precipitated and analysed by LC–MS/MS.

For the identification of CHMP4C interactors, the raw MS data were analysed using the MASCOT search engine (http://www.matrixscience.com). Peptides were searched against the UniProt human sequence database, and the following search parameters were employed: enzyme specificity was set to trypsin, a maximum of two missed cleavages were allowed, carbamidomethylation (Cys) was set as a fixed modification, while oxidation (Met), phosphorylation (Ser, Thr and Tyr) and ubiquitylation (Lys) were considered as variable modifications. Peptide and MS/MS tolerances were set to 25 parts per million (ppm) and 0.8 Da, respectively.

### 4.8. GFP pull-down assay

Cells stably expressing MKLP1::GFP (a gift from M. Mishima, University of Warwick, UK) were transfected with plasmids encoding Flag-tagged CHMP4C-WT or the two mutant variants S210A and STripleA and synchronized in telophase as described in the previous section. Cells were then collected and stored at −80°C. The cell pellet was resuspended in 0.5 ml of extraction buffer (50 mM HEPES pH 7.5, 100 mM KAc, 50 mM KCl, 2 mM MgCl_2_, 2 mM EGTA, 0.1% NP-40, 5 mM DTT, 5% glycerol and Roche Complete Protease Inhibitors) and homogenized using a high-performance disperser (Fisher). Homogenates were centrifuged at 2000 r.p.m. at 4°C in an Eppendorf 5417R centrifuge for 10 min and supernatants transferred into new tubes. GFP-Trap-MADynabeads (25 µl; Chromotek) was added to the supernatants and incubated for 2 h on a rotating wheel at 4°C. Beads were then washed five times for 5 min in 0.5 ml of extraction buffer. Proteins were eluted from beads with 0.5 ml of elution buffer (0.5 M NH_4_OH and 0.5 mM EDTA pH 8.0), lyophilized and resuspended in Laemmli SDS–PAGE sample buffer (Sigma) and boiled for 10 min. Proteins were separated on an SDS–PAGE gel, transferred onto a PVDF membrane and probed to detect GFP and Flag antibodies.

### 4.9. *In vitro* binding assay

DNA fragments coding for CHMP4C and MKLP1 fragments were generated by PCR and cloned into pDEST15 (Thermo), using Gateway technology to express N-terminal GST-tagged polypeptides in *E. coli*. The GST-tagged products were then purified, using glutathione sepharose 4B according to manufacturer's instruction (GE Healthcare). [^35^S] Methionine-labelled CHMP4C and MKLP1 polypeptides were prepared from corresponding PCR products amplified, using primers harbouring a T7 promoter and then transcribed and translated *in vitro* using the TnT T7 Quick Coupled Transcription/Translation System (Promega) in the presence of [^35^S] methionine (Perkin Elmer). The binding reaction contained 150 mM NaCl and subsequent washes varied from 150 mM to 1 M NaCl. GST pull down assays were carried out as described previously [37].

## Supplementary Material

RSOB-16-0248.R1 - Supplementary text and figures

## Supplementary Material

Figure S1

## Supplementary Material

Figure S2

## Supplementary Material

Figure S3

## Supplementary Material

Figure S4

## Supplementary Material

Figure S5

## Supplementary Material

Figure S6

## Supplementary Material

Table S1

## Data accessibility

The MS datasets supporting this article have been uploaded as part of the electronic supplementary material.
