# Supplementary material for: Coordinated regulation of the ESCRT-III component CHMP4C by the chromosomal passenger complex and centralspindlin during cytokinesis
Source: Open Biol. 2016 Oct 26;6(10):160248. doi: 10.1098/rsob.160248 (PMC5090064; doi:10.1098/rsob.160248)
Supplement: RSOB-16-0248.R1 - Supplementary text and figures [file rsob160248supp1.docx]

**Capalbo et al.**

**Supplementary Information**

**Supplementary Figure Legends**

**Figure S1**. CHMP4C can spontaneously assemble into spiral filaments. (a) Low (left) and high (right) magnification AFM images of mica incubated with CHMP4C. Scale bars are: 200 nm (left) and 100 nm (right). (b) Low (left) and high (right) magnification AFM images of mica incubated with the mini-CPC. Scale bars are: 200 nm (left) and 20 nm (right). (c) Low (left) and high (right) magnification AFM images of mica incubated with CHMP4C and the mini-CPC. Scale bars are: 200 nm (left) and 20 nm (right). In each low magnification image the red rectangle marks the area magnified on the right. A height bar relative to the high magnification image is shown on the far right.

**Figure S2.** Incubation with CHMP4C interferes with the apparent height of the lipid bilayer membrane. (*a*) Tridimensional AFM image of a plain lipid bilayer. (*b*) Height relative to the mica surface is plotted along the dotted red line shown in (*a*). (*c*) Tridimensional AFM image of a lipid bilayer incubated with CHMP4C. Protein and fragments of lipid bilayer can be seen in the gaps of the membrane. (*d*) Height relative to the mica surface is plotted along the dotted red line shown in (*c*).

**Figure S3**. Aurora B interacts directly with CHMP4C. (*a*) Schematic diagram illustrating the structure of CHMP4C. The CHMP4C α-helices are marked at the top. (*b*) The GST::CHMP4C protein fragments shown at the top and GST alone were incubated with *in vitro* translated and radiolabelled Aurora B and then pulled down using glutathione beads. The Ponceau S staining of the protein loading is shown at the bottom and the numbers on the left indicate the sizes (in kDa) of the molecular mass markers.

**Figure S4.** Characterization and selection of cell lines stably expressing wild type and phospho-dead CHMP4C variants. Different monoclonal HeLa Kyoto cell lines expressing either wild-type (WT), S201A, or StripleA CHMP4C mutants were fixed and stained to detect DNA and tubulin and the number of multinucleate cells was counted and plotted. More than 800 cells were counted. The following cell lines were selected for future experiments because they showed the lowest cytokinesis defects: clone 26 for CHMP4C-WT, clone 8 for CHMP4C-S210A, and clone 1-18 for CHMP4C-StripleA,

**Figure S5**. Validation of the phospho-specific CHMP4C antibodies. (*a*) Schematic diagram illustrating the structure of CHMP4C. The CHMP4C α-helices are marked at the top. (*b*) GST-tagged wild type (WT) and mutants versions of the C-terminal half of GST::CHMP4C (aa 122-233) and GST alone were purified from bacteria and incubated with (+) or without (-) Aurora B in the presence of ATP. The reactions were separated by SDS-PAGE and probed with antibodies specific for either tri- or mono-phospho CHMP4C. The Ponceau S staining of the protein loading is shown at the bottom and the numbers on the left indicate the sizes (in kDa) of the molecular mass markers. Please note that mono- and tri-phospho CHMP4C both detected a signal when WT-CHMP4C is phosphorylated by Aurora B *in vitro* (lane 4) most likely because this sample contains a mix of different phosphorylated CHMP4C peptides (i.e. phosphorylated only at S210 and phosphorylated at all three serine residues, S210, S214 and S215). (*c*) HeLa Kyoto cells were fixed and stained to detect tri-phospho CHMP4C (red), tubulin (green), and DNA (blue) in the presence of either the peptide used to generate the tri-phospho CHMP4C antibody or an unrelated peptide as control. Insets show 2X magnification of the midbody. Scale bars, 10 μm.

**Figure S6**. Localization of tri-phospho CHMP4C in metaphase. HeLa Kyoto cells were fixed and stained to detect tri-phospho CHMP4C (red), tubulin (green or blue), DNA (blue) and proteins marking either the centromere (i.e. Borealin and CREST [1]), or the kinetochore (i.e. Blinkin [2]). Insets show 2X magnification (rotated 90° in the top three panels) of the metaphase plate. Scale bars, 10 μm.

**Supplementary Video Legends**

**Video S1.** CHMP4C alone has membrane remodeling activity. This movie shows CHMP4C activity on a lipid bylayer on mica surface. See Figure 1 for details. Frames were captured at 25 seconds intervals. Playback is 2 frames per second.

**Video S2.** CPC core inhibits CHMP4C binding and membrane remodeling activity on the lipid bilayer. This movie shows a lipid bilayer that not change over time. See Figure 1 for details. Frames were captured at 25 seconds intervals. Playback rate is 2 frames per second.

**Video S3.** Aurora B phosphorylation inhibits the membrane remodeling activity of CHMP4C on the lipid bilayer, but not its binding. This movie shows binding of CHMP4C to the curved bilayer edge, but not remodeling activity. See Figure 1 for details. Frames were captured at 25 seconds intervals. Playback rate is 2 frames per second.

**Video S4.** Aurora B bound to CHMP4C partially inhibits the membrane remodeling activity of CHMP4C on the lipid bilayer. This video shows some membrane gaps closing and then collapsing. Frames were captured at 25 seconds intervals. Playback rate is 2 frames per second.

**Supplementary Table S1**

Lists of proteins identified by MS from the pull downs of Flag alone and Flag::CHMP4C (wild type) from cell synchronized in telophase. The proteins that were specific for the Flag::CIT-K pull down are listed in the first worksheet, while the full lists of proteins identified after either the Flag pull down or the Flag::CHMP4C pull down are listed in the second and third worksheet, respectively.

**References**

1. Tan, E. M., Rodnan, G. P., Garcia, I., Moroi, Y., Fritzler, M. J., Peebles, C. 1980 Diversity of antinuclear antibodies in progressive systemic sclerosis. Anti-centromere antibody and its relationship to CREST syndrome. *Arthritis Rheum*. **23**, 617-625.

2. Kiyomitsu, T., Obuse, C., Yanagida, M. 2007 Human Blinkin/AF15q14 is required for chromosome alignment and the mitotic checkpoint through direct interaction with Bub1 and BubR1. *Dev Cell*. **13**, 663-676. (10.1016/j.devcel.2007.09.005)
