# Supplementary figures and images for: Coordinated regulation of the ESCRT-III component CHMP4C by the chromosomal passenger complex and centralspindlin during cytokinesis
Source: Open Biol. 2016 Oct 26;6(10):160248. doi: 10.1098/rsob.160248 (PMC5090064; doi:10.1098/rsob.160248)

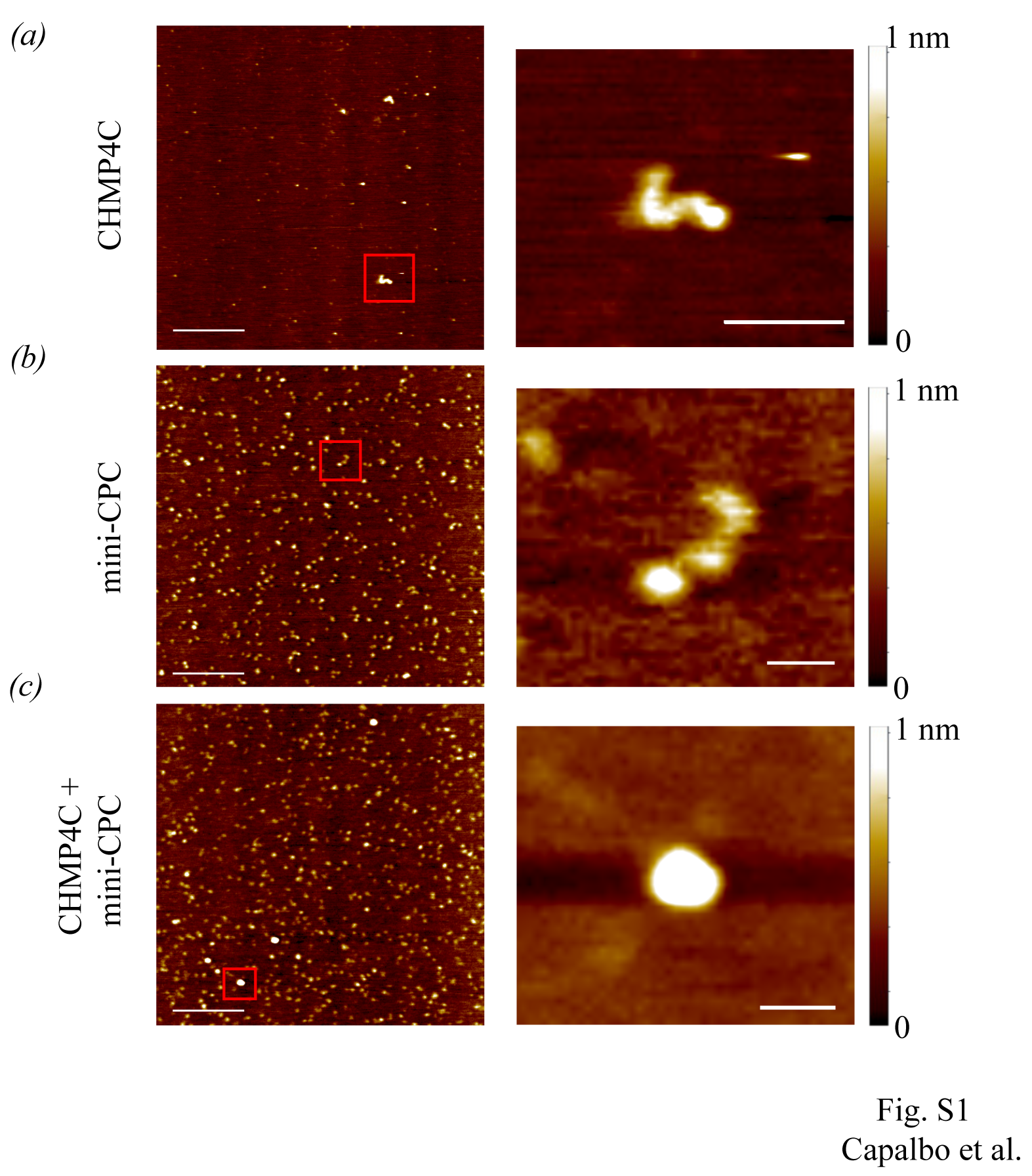

Supplement: Figure S1 [file rsob160248supp2.tif]

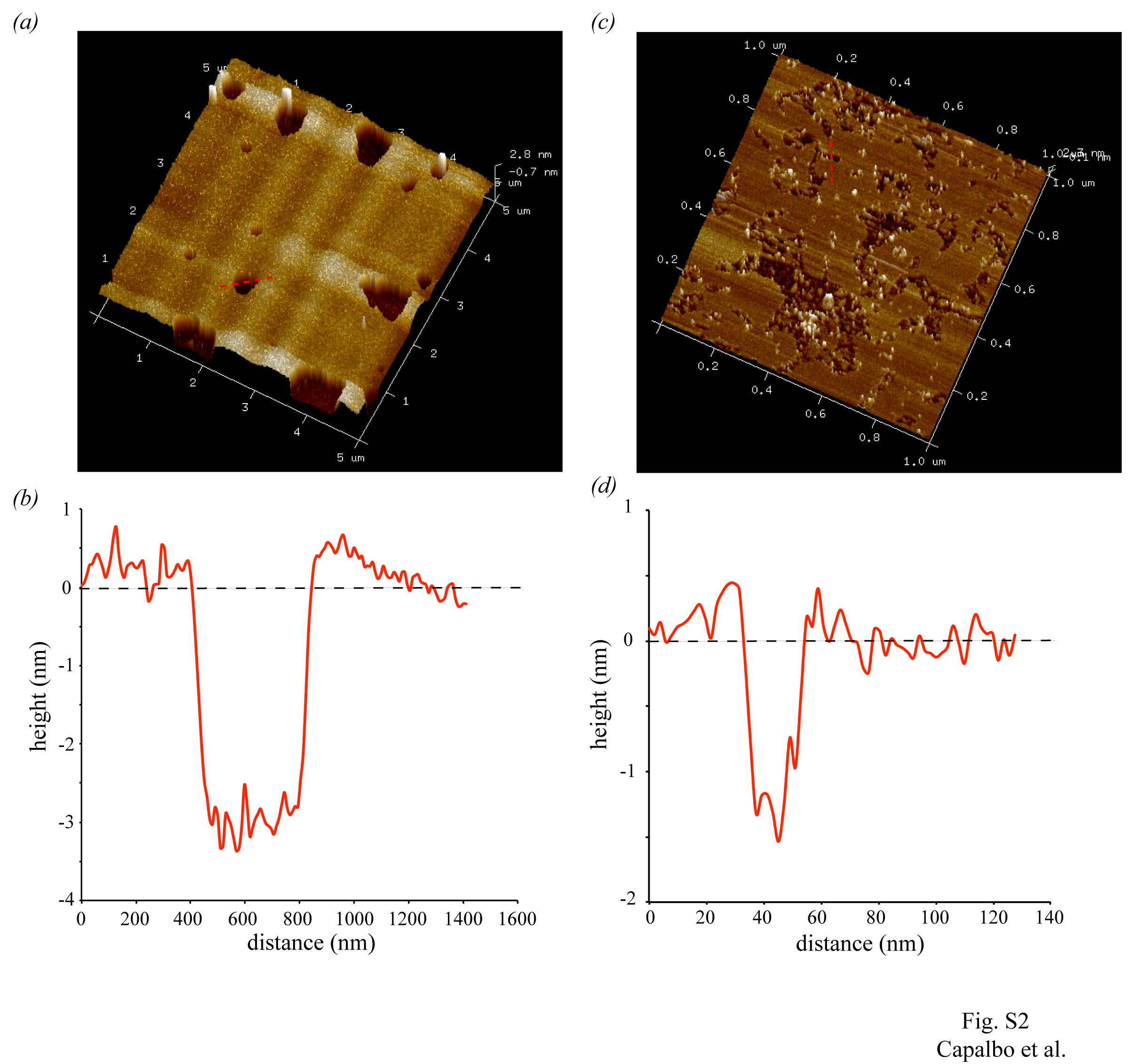

Supplement: Figure S2 [file rsob160248supp3.tif]

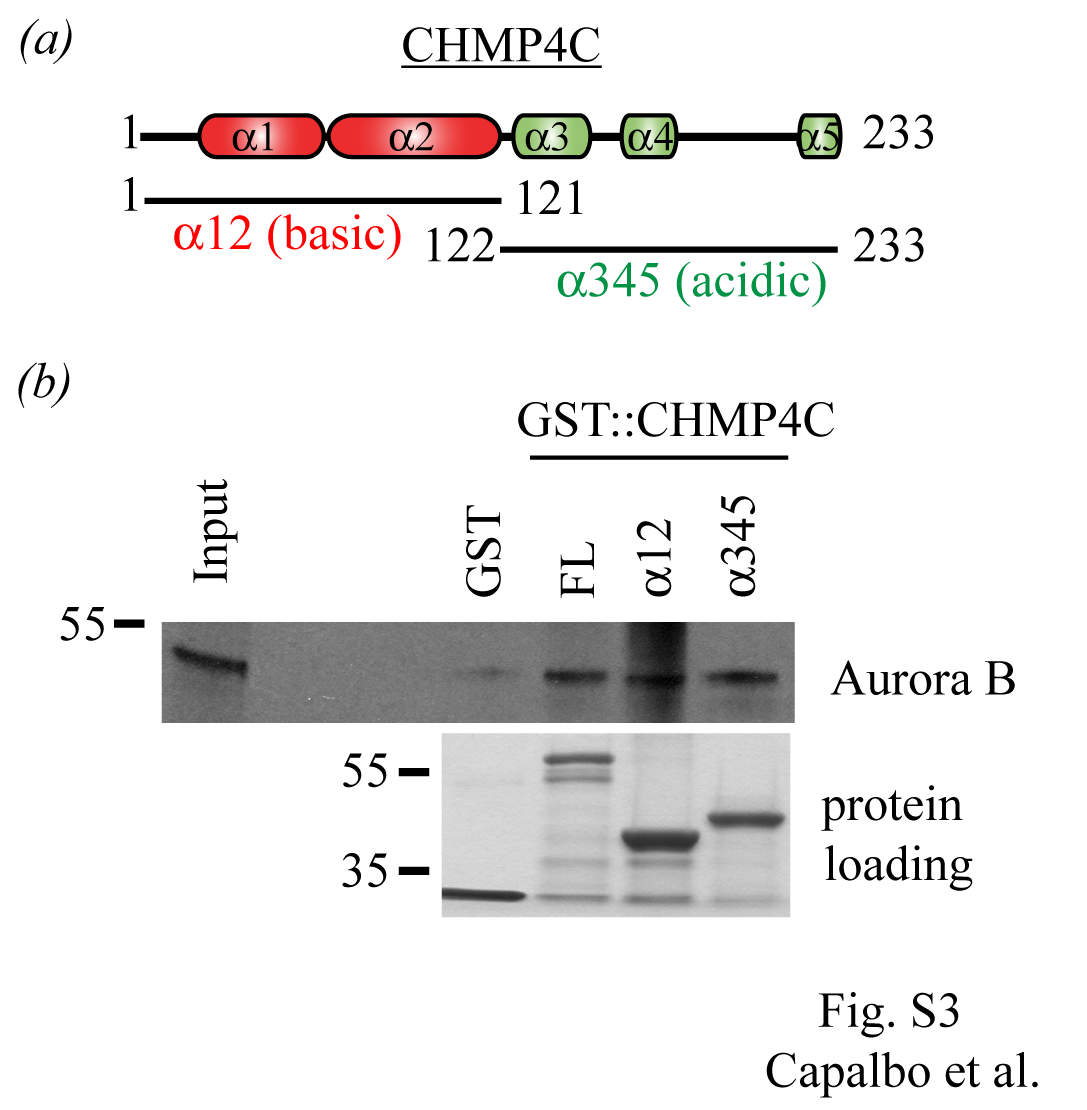

Supplement: Figure S3 [file rsob160248supp4.tif]

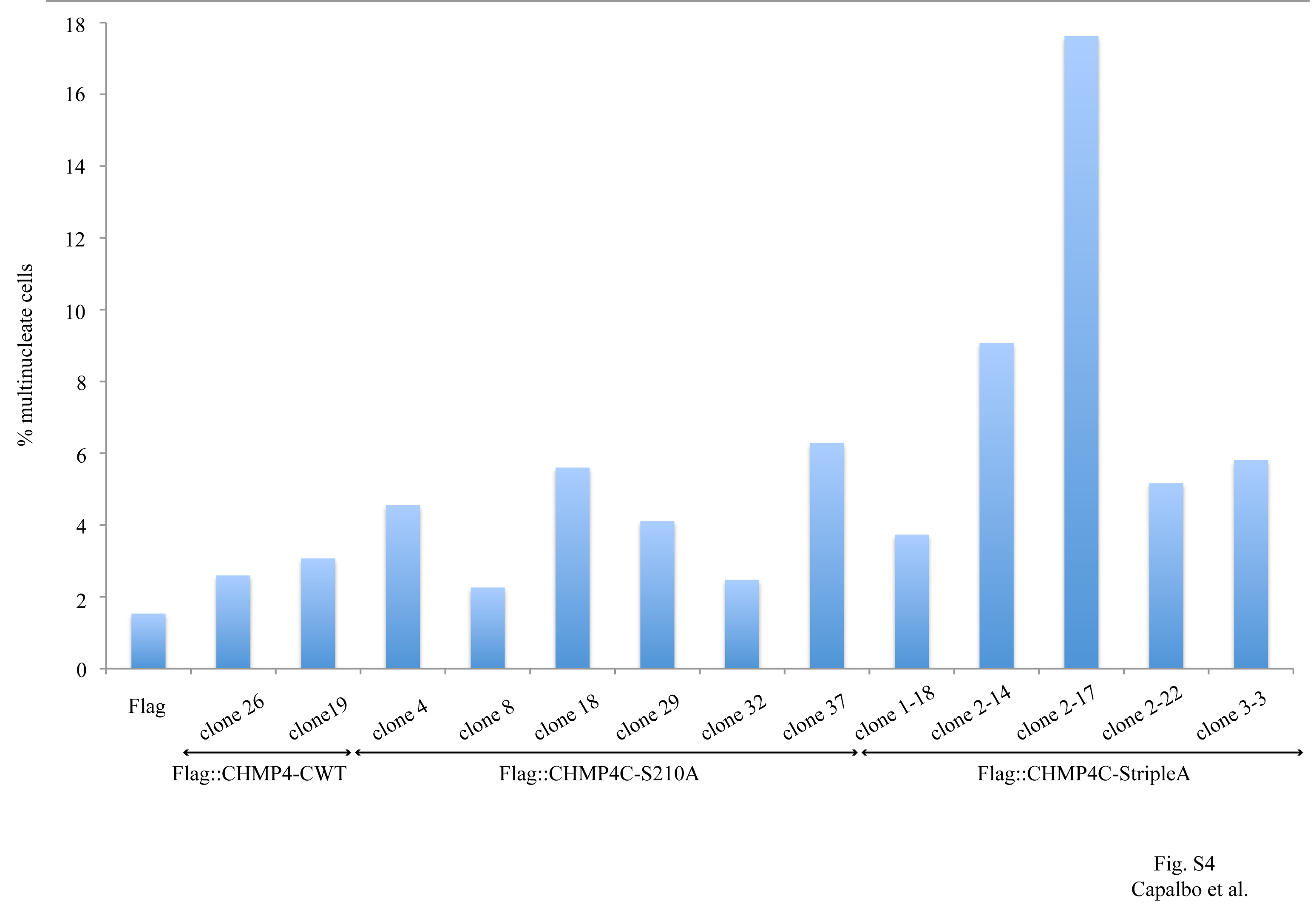

Supplement: Figure S4 [file rsob160248supp5.tif]

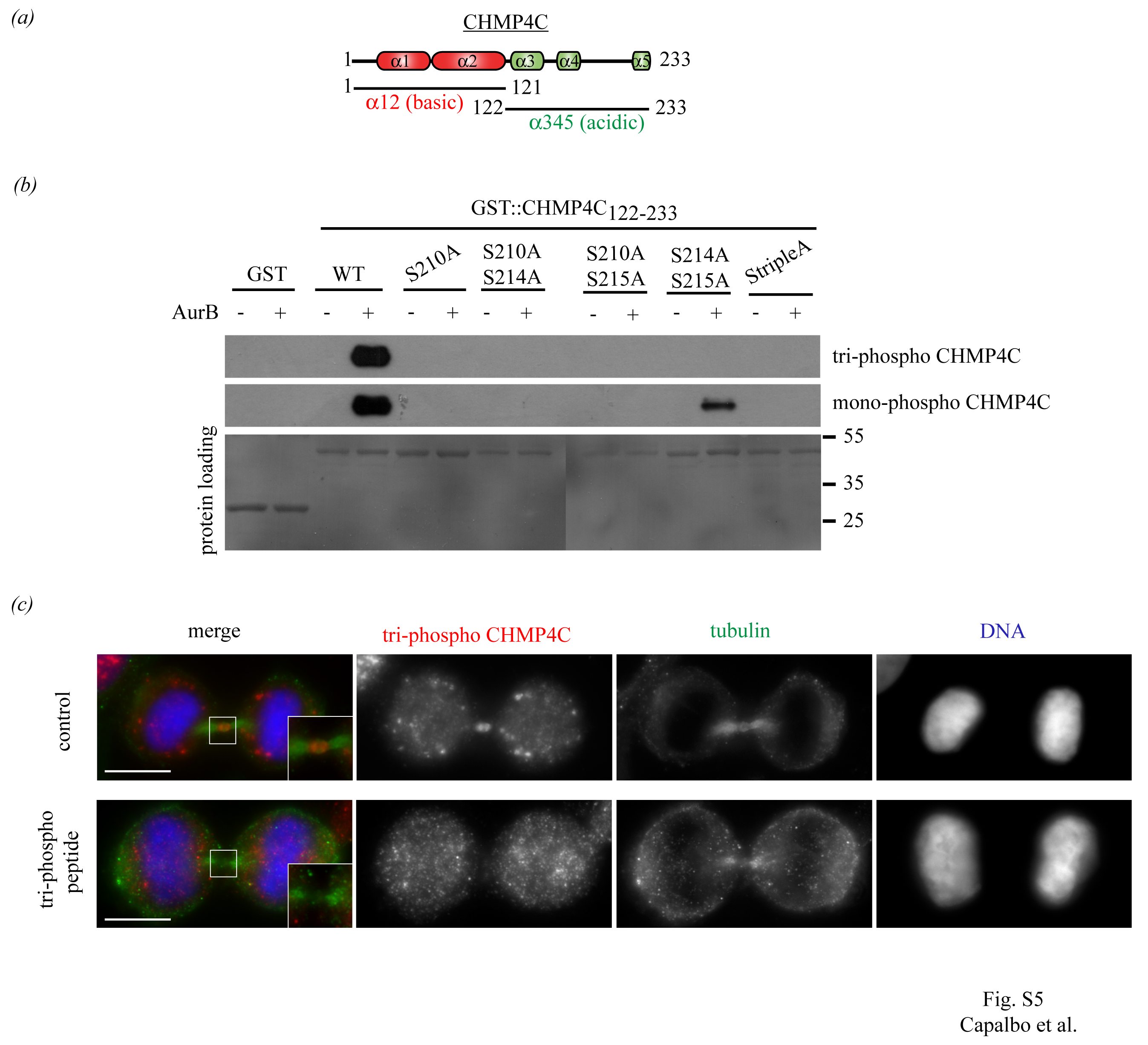

Supplement: Figure S5 [file rsob160248supp6.tif]

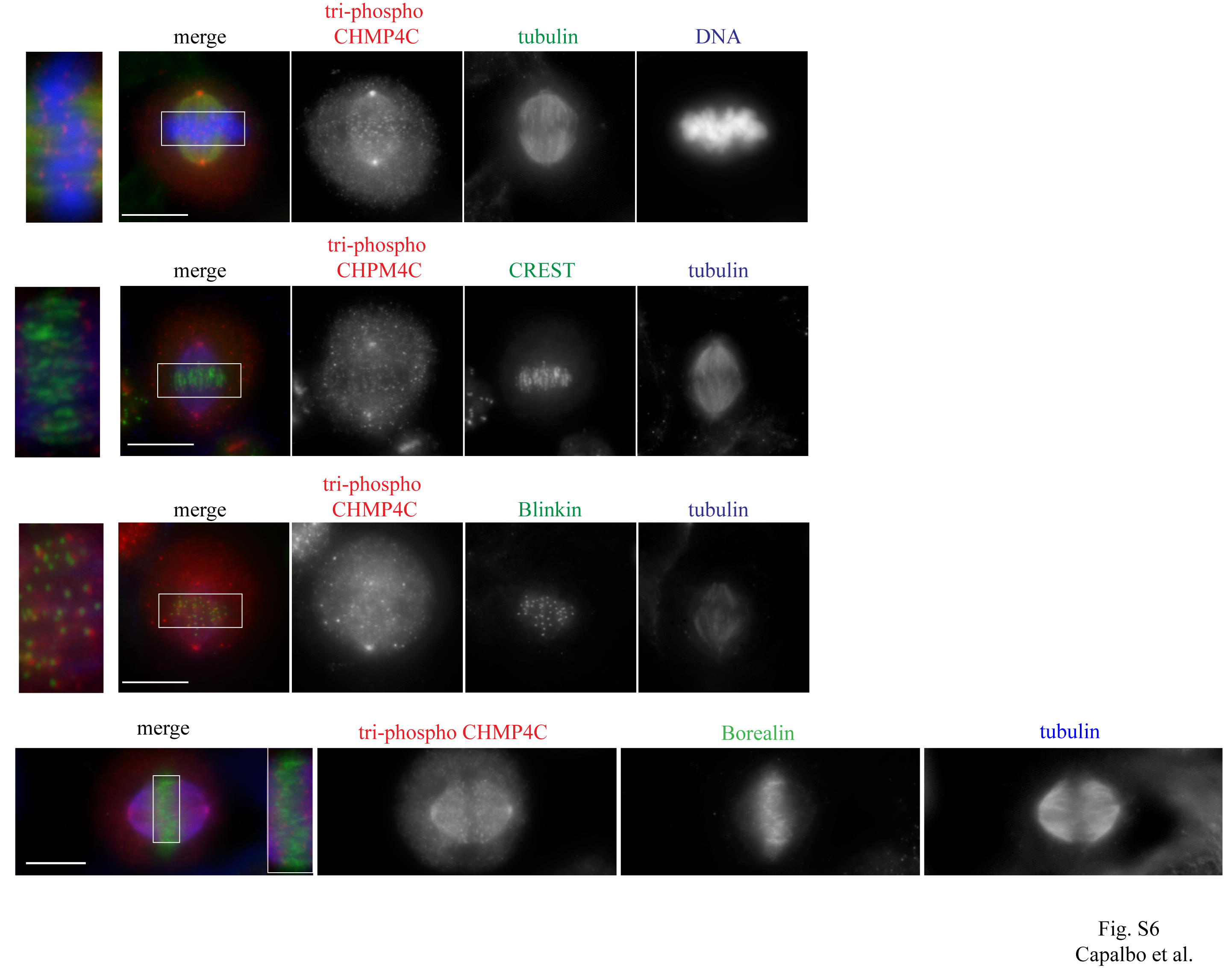

Supplement: Figure S6 [file rsob160248supp7.tif]
